# The Potential Role of Hypothalamic Phospholipid Liposomes in the Supportive Therapy of Some Manifestations of Post-COVID-19 Condition: Myalgic Encephalomyelitis/Chronic Fatigue Syndrome (ME/CFS) and Brain Fog

**DOI:** 10.3390/jcm12175478

**Published:** 2023-08-23

**Authors:** Francesco Menichetti

**Affiliations:** Italian Group for Antimicrobial Stewardship (GISA), 27100 Pisa, Italy; menichettifrancesco@gmail.com

**Keywords:** post-COVID-19 condition, long COVID, post-COVID syndrome, neuroendocrine disorders, chronic fatigue, brain fog, phospholipids, phospholipid liposome, phosphatidylserine

## Abstract

Post-COVID-19 condition (commonly known as Long COVID) is a heterogeneous clinical condition in which Myalgic Encephalomyelitis/Chronic Fatigue Syndrome (ME/CFS) and brain fog stand out among the different clinical symptoms and syndromes. Cerebral metabolic alterations and neuroendocrine disorders seem to constitute an important part of the pathophysiology of Post-COVID-19 condition (PCC). Given the substantial lack of specific drugs and effective therapeutic strategies, hypothalamic phospholipid liposomes, which have been on the market for several years as adjuvant therapy for cerebral metabolic alterations resulting from neuroendocrine disorders, might represent a potential option in an overall therapeutic strategy that aims to control PCC-associated symptoms and syndromes. Their pharmacological mechanisms and clinical effects strongly support their potential effectiveness in PCC. Our initial clinical experience seems to corroborate this rationale. Further controlled clinical research is warranted in order to verify this hypothesis.

## 1. Introduction

By April 2023, the COVID-19 pandemic in Italy caused over 25 million documented infection cases and about 190,000 deaths [1]. Beyond the devastating impact on hospital services, which, in the first pandemic phase (2020–2021), were engaged almost exclusively in dealing with COVID-19, and a serious delay in taking care of other pathologies, despite now having the epidemic wave under control, thanks to vaccines and wise behavior, a new pathological entity has emerged that seems to be a direct consequence of the SARS-CoV-2 infection: Post-COVID-19 condition (commonly known as Long COVID) [2,3,4,5]. We analyze the potential role of hypothalamic phospholipid liposomes as an adjuvant therapy for some important manifestations of Post-COVID-19 condition, such as Myalgic Encephalomyelitis/Chronic Fatigue Syndrome and brain fog, based on the current knowledge on Post-COVID-19 condition and hypothalamic phospholipid liposomes’ pharmacology.

## 2. Definition and Epidemiology of Post-COVID-19 Condition

Many definitions have been adopted to describe this complex of symptoms and syndromes that may follow the COVID-19 infection [2,3]. The term “Post-COVID-19 condition” (PCC) has been adopted by the World Health Organization (WHO) and the Center for Disease Control (CDC), and it refers to a wide range of sequelae that compromise the physical and mental health of some patients and that occur after SARS-CoV-2 infection [3,6]. PCC is referred to by a wide range of names, including Long COVID, Post-acute COVID-19, Long-term effects of COVID, Post-acute COVID syndrome, Chronic COVID, Long-lasting COVID, Late sequelae, Post-acute sequelae of SARS-CoV-2 infection (PASC).

The CDC indicates that PCC is present if recovery does not occur within 4 weeks after the acute phase, although many patients may continue to improve within 12 weeks [6,7]. After 12 weeks, persistent illness becomes more likely [7]. In fact, the WHO defines PCC as a condition occurring 3 months after acute infection, lasting for at least 2 months [3]. However, the 4-week threshold allows a rapid identification and treatment of PCC [8]. The CDC uses the 4-week period to define PCC and emphasizes the importance of initial clinical evaluation and supportive care during the initial 4–12 weeks after acute COVID-19 [7].

The prevalence of PCC is difficult to estimate, with studies reporting a range from 5 to 30% [7,9]. The variety of symptoms or conditions studied, the time criteria used (from three weeks to many months after SARS-CoV-2 infection), the population studied (outpatient vs. hospitalized patients), and the method used to evaluate symptoms and conditions (e.g., self-reporting vs. electronic health record database) are a few factors that contribute to these wide-ranging prevalence estimates [7,10,11,12]. Recent data have shown that up to 18% of SARS-CoV-2-infected individuals are affected by PCC two years after infection, even though a decrease in the severity of symptoms and health impairment may be seen over time [13]. CDC data demonstrate that women, bisexual and transgender adults, as well as adults with disabilities are more likely to have PCC [14].

## 3. Clinical Symptoms and Syndromes

The PCC manifestations comprise a long list of symptoms referring to different apparatuses (Table 1). The core symptoms include fatigue, brain fog, breathlessness, anosmia, and mental health problems among others [15].

The heterogeneity of this condition is further demonstrated by its different patterns of onset, such as [7]:

- Persistent symptoms and conditions that begin at the time of acute COVID-19 illness;
- Signs, symptoms, or conditions of new onset following asymptomatic illness or a period of improvement or remission of acute symptoms;
- Evolution of symptoms and conditions, which include some persistent symptoms (e.g., shortness of breath) with the addition of new symptoms (e.g., cognitive difficulties);
- Worsening of pre-existing symptoms or conditions.

Some manifestations may share similarities with other post-infectious syndromes, such as Myalgic Encephalomyelitis/Chronic Fatigue Syndrome (ME/CFS), Fibromyalgia (FM), post-treatment Lyme diseases syndrome (PTLDS), Postural Orthostatic Tachycardia Syndrome (POTS) and other forms of dysautonomia, or Mast Cell Activation Syndrome (MCAS). Some of these conditions have also been described in patients with Severe Acute Respiratory Syndrome (SARS) and Middle East Respiratory Syndrome (MERS), two other life-threatening diseases resulting from coronavirus infections [7,16].

A wide range of other symptoms, new or already present, can occur in people who have suffered from SARS-CoV-2 acute disease of varying degrees, including the mild asymptomatic form [10,12]. These symptoms may overlap with multiple organ complications or the effects of treatment or hospitalization. It can be difficult to distinguish the symptoms of PCC from those that have other causes. It is, therefore, always necessary to consider alternative diagnoses in order to avoid serious diagnostic errors, in cases of patients presenting with symptoms such as dyspnea, chest pain, neurological disorders, etc. [17].

**Table 1:** Post-COVID syndromes and their clinical manifestations (adapted from [17,18]).

Post-COVID Syndrome	Clinical Manifestations	Comment
Post-COVID fatigue syndrome	Profound fatigue, post-exertion malaise, and/or poor resistance	Rule out causes like anemia, electrolyte imbalance, hypothyroidism.
Post-COVID cardio-respiratory syndrome	Cough, dyspnea or increased fatigue, low-grade fever, chest pain, orthostatic hypotension, palpitations, and tachycardia	Sudden worsening of dyspnea: consider tension pneumothorax, pulmonary embolism, coronary artery disease, or heart failure.
Post-COVID neuro-psychiatric syndrome	Headaches, anosmia or dysgeusia, cognitive impairment or “brain fog”, depression and other mood changes, paresthesia, insomnia and other sleep difficulties, dizziness	If acute onset neurological symptoms present, also consider vasculitis, thrombosis, or demyelination. Properly evaluate post-COVID psychological problems.
Post-COVID gastro-intestinal syndrome	Abdominal discomfort, diarrhea, constipation, vomiting	GI symptoms can be a sequalae of the disease or therapy-related side effects.
Post-COVID hepato-biliary syndrome	Nausea, jaundice, liver function test alterations	Drugs used in the treatment of COVID-19 can cause hepatic impairment.
Post-COVID musculo-skeletal syndrome	Arthralgia, myalgia, muscle weakness	Causes include COVID-19 disease, prolonged ICU care, neurological problems, myopathy, or electrolyte imbalance. Usually subside during follow up. Inflammatory arthralgia must be differentiated from other causes like Systemic Lupus Erythematosus, Rheumatoid Arthritis.
Post-COVID thromboembolic syndrome	Depending upon the vascular territory of involvement, dyspnea in Pulmonary Embolism, chest pain in Coronary Artery Disease, and limb weakness and neurological deficit in stroke	Early diagnosis and treatment are lifesaving. Follow the standard treatment protocol.
Post-COVID multisystem inflammatory syndrome/post-COVID autoimmune syndrome	Fever, gastrointestinal symptoms, rash, chest pain, palpitations	Elevated levels of markers of inflammation.
Post-COVID genito-urinary symptoms	Proteinuria, hematuria, development of kidney injury, menstrual cycle irregularities, erectile dysfunction	COVID-19 may predispose surviving patients to chronic kidney disease, independently of clinically apparent acute kidney injury (AKI). Therefore, post-acute COVID-19 care should include close attention to kidney function.
Post-COVID dermatological syndrome	Vesicular, maculopapular, urticarial, or chilblain-like lesions on the extremities (COVID toe)	

PCC has a substantial impact on ability to work and activities of daily living [7,15]. Patients suffering from PCC show high levels of functional impairment and low health-related quality of life. These results seem to be comparable to, or even worse than, those of patients suffering from diseases such as Parkinson’s disease, stroke, or advanced/metastatic cancers [15]. Fatigue appears to be the symptom most strongly associated with functional impairment, causing significant impact on the patients’ ability to work and care for others [15].

### 3.1. Myalgic Encephalomyelitis/Chronic Fatigue Syndrome (ME/CFS)

This condition has been already described as a consequence of viral infections, (e.g., after infectious mononucleosis, especially in patients who present the prolonged persistence of anti-EBV IgM); ME/CFS is one of the most prevalent and most disabling syndromes in PCC [19,20,21,22].

According to the National Academy of Medicine, the diagnosis of ME/CFS requires the concomitant presence of the following three symptoms [19]:

1. Substantial reduction or alteration of employment, educational, social, or personal capacities that persists for more than 6 months and is accompanied by asthenia, often profound, of new or recent onset (not pre-existing), that is not the result of continuous excessive effort and is not effectively alleviated by rest.
2. Post-exertion malaise.
3. Non-restorative sleep.

The presence of cognitive impairment and/or orthostatic intolerance is also required for the diagnosis [19]. Symptoms of post-exertion malaise, non-restorative sleep, and cognitive impairment should be at least of moderate intensity and present at least 50% of the time during a 6-month period to make the diagnosis of ME/CFS [19].

**Figure 1:**
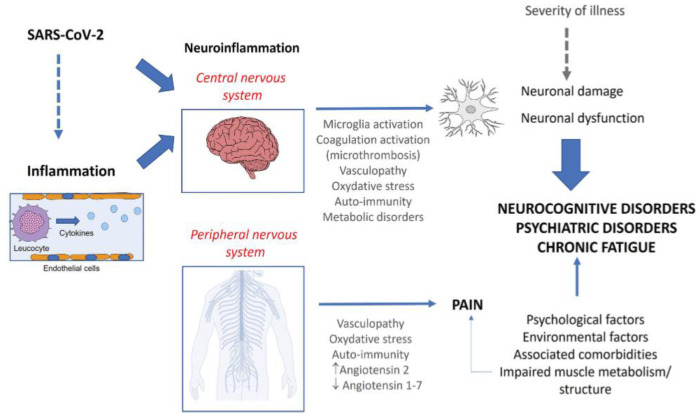
Putative pathophysiological mechanisms involved in Post-COVID-19 condition [23].

The possible mechanisms that cause Post-COVID-19 fatigue comprise a wide range of central, peripheral, and psychological factors [20,21,22,23]. Chronic inflammation in the brain, as well as in the neuromuscular junctions, can result in chronic fatigue [23]. Damage and atrophy of skeletal muscle fibers have been proposed to play a role as well [22,23] (Figure 1).

### 3.2. Cognitive Impairment

Cognitive symptoms are a major feature of PCC [16]. Often referred to by patients as “brain fog”, cognitive impairment in PCC affects different domains, with memory problems and attention difficulties being its most notable manifestations [13,24]. Activities of daily living are frequently impacted by cognitive impairment in PCC, and research suggests that it may be as debilitating as 10 years of cognitive aging [16].

Several underlying mechanisms of cognitive impairment have been hypothesized in PCC: hypometabolic activity in various brain areas or a reduced inhibitory activity of GABA, but also neuroinflammatory phenomena with cerebral microstructural modifications and vascular disorders [21,23,25,26].

**Figure 2:**
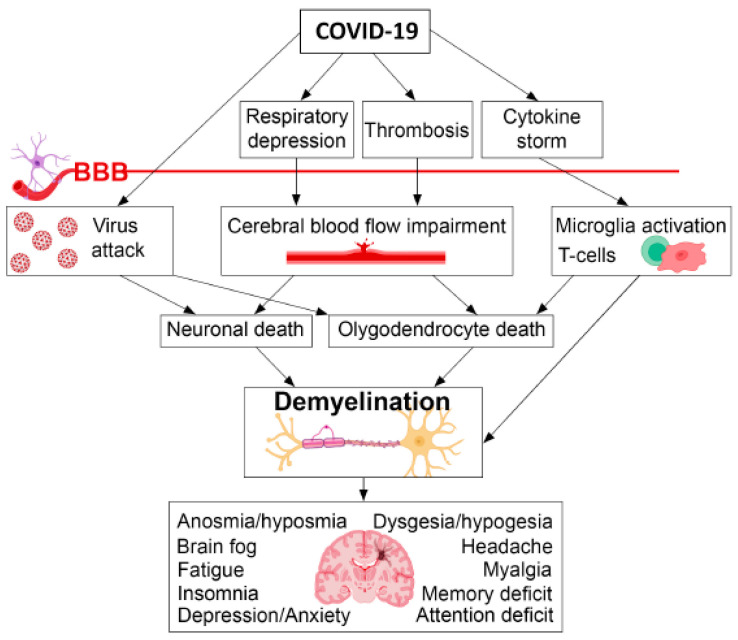
A schematic representation of the relationships between COVID-19, demyelination, and neuropsychiatric sequalae (BBB—Blood Brain Barrier) [27].

One explanation of the multiple neuro-cognitive symptoms of PCC may be demyelination generated by the concomitant action of viral replication, alterations of cerebral microcirculation, and activation of microglia T cells (Figure 2) [27]. The neuroradiological findings are, however, modest and sporadic because a very limited number of studies have used highly sensitive techniques for myelin quantification [27].

Another hypothesis that received attention because it might offer either a comprehensive pathophysiological understanding of the mechanism underlying several manifestations of PCC or some therapeutic perspectives refers to the generation of amyloid fibrin micro-clots in the vascular system [28]. This hypothesis, supported by an elegant although isolated scientific report, has however generated the uncontrolled use of expensive treatments whose real effectiveness is at least doubtful: blood washing and triple anticoagulation therapy [29]. Many patients turn to private facilities in Europe to undergo apheresis procedures, spending a lot of money for a procedure without clear evidence of efficacy [29].

The National Institute of Health has committed more than USD 1 billion to post-COVID-19 research, and the WHO is coordinating global efforts [30]. Although it is challenging to fully understand and address the post-infectious sequelae of SARS-CoV-2, doing so will increase the likelihood of finding effective therapies [30].

In the meantime, several drugs and nutrients have been proposed and tested to control PCC clinical manifestations (e.g., low-molecular-weight heparin, vitamin D, etc.) [31,32,33]. Nevertheless, we are dealing with a substantial lack of specific drugs and effective therapeutic strategies [15]. It is, therefore, necessary to try to help every single patient by also considering the potential role of preparations that have been on the market for several years, with an excellent safety profile, which can be taken into consideration in an overall therapeutic strategy that aims to control PCC-associated symptoms and syndromes.

## 4. The Potential Role of Hypothalamic Phospholipid Liposomes in Post-COVID-19 Condition

In COVID-19, important variations in sphingolipids and glycerophospholipids have been described: the increase in the blood level of specific compounds seems to be correlated with the degree of disease severity [34,35,36,37]. In particular, elevated levels of phosphatidylcholine (PC) correlate with a less severe form of COVID-19, and this could be useful both as a prognostic marker and as a potential therapeutic intervention [37]. Furthermore, alterations of the phospholipid metabolism as well as the phospholipid composition of cellular structures (such as the mitochondria of microglia) have also been reported in the literature [38,39]. On the other hand, various complex mechanisms underly the pathophysiology of PCC including cerebral metabolism alterations and neuroendocrine disorders [40]. Indeed, recent findings that the SARS-CoV-2 spike protein can bind to the receptors of the neuroendocrine system shed light on the neuroendocrine involvement in COVID-19 [41]. Additionally, the levels of copeptin, a neuroendocrine biomarker of the stress response by Hypothalamic–Pituitary axis, correlate with COVID-19 severity [42]. COVID-19 infection alters the Hypothalamic–Pituitary–Adrenal (HPA) axis due to direct viral infection of hypothalamic structures or the effect of pro-inflammatory cytokines [43,44]. Finally, among the heterogenous clinical manifestations of PCC, ME/CFS is a syndrome characterized by the presence of neuroendocrine disorders as part of its pathophysiological and clinical features [45,46].

In this view, taking into account the alterations of the phospholipid metabolism, as well as the importance of the neuroendocrine disorders in the pathophysiology and the clinical manifestations of PCC, a medicine containing a mixture of hypothalamic phospholipids (Liposom Forte^®^) indicated as “adjuvant therapy of cerebral metabolic alterations resulting from neuroendocrine disorders” elicits particular interest [47,48]. Liposom Forte^®^ is a mixture of hypothalamic phospholipids in the form of liposomes. It is extracted from porcine brain, and its major components are phosphatidylcholine (PC), phosphatidylethanolamine (PE), and phosphatidylserine (PS), representing all together about 90% of the total phospholipids of the mixture [47]. Hypothalamic phospholipid liposomes reach the central nervous system where they exert different effects by influencing the physicochemical and structural properties of the neural membrane, as well as by affecting its function and that of the related cellular structures [47,48]. Liposom Forte^®^ has shown an excellent safety profile during its long-time presence on the market [47]. Its mechanism of action, as well as the clinical evidence on its efficacy, offers a strong rationale for the use of hypothalamic phospholipid liposomes in PCC.

### 4.1. Pathophysiological Mechanisms in Post-COVID-19 Condition and the Pharmacology of Hypothalamic Phospholipid Liposomes

**Figure 3:**
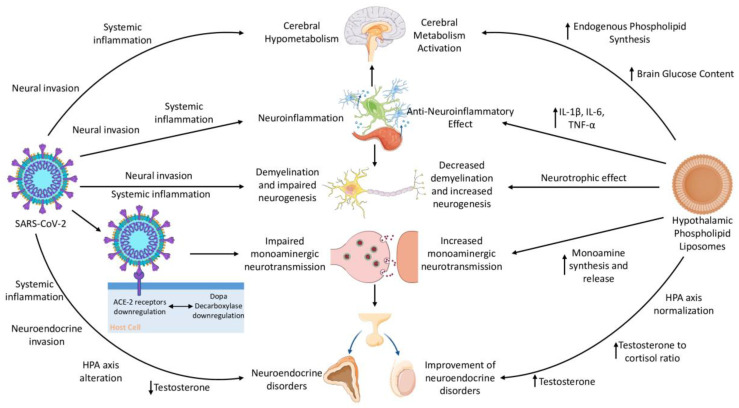
A schematic representation of Post-COVID-19 condition’s pathophysiology and the relevant hypothalamic phospholipid liposomes’ mechanism of action (ACE-2—Angiotensin I Converting Enzyme 2; IL-1β—Interleukin 1β; IL-6—Interleukin 6; TNF-α—Tumor Necrosis Factor α; HPA axis—Hypothalamic–Pituitary–Adrenal axis). Parts of the figure were drawn by using pictures from Servier Medical Art. Servier Medical Art by Servier is licensed under a Creative Commons Attribution 3.0 Unported License (https://creativecommons.org/licenses/by/3.0).

There are many mechanisms by which hypothalamic phospholipid liposomes could benefit patients with PCC, especially those affected by ME/CFS and brain fog. We explore here the pathophysiological mechanisms of PCC that may be the target of some of the pharmacological effects of hypothalamic phospholipid liposomes (Figure 3).

#### 4.1.1. The Monoaminergic Hypothesis

Monoaminergic neurotransmission alterations have been proposed as a potential pathophysiological mechanism for the neuropsychiatric manifestations of PCC [49]. This is suggested by a significant link between Angiotensin I Converting Enzyme 2 (ACE2, encoding the main receptor to SARS-CoV-2) and Dopa Decarboxylase (DDC, encoding the enzyme that catalyzes the biosynthesis of dopamine, noradrenaline, and serotonin). Indeed, the gene exhibiting the most statistically significant co-expression link with ACE2 is DDC [49]. The co-expression and co-regulation of ACE2 and DDC are corroborated by findings such as high ACE2 expression in dopaminergic neurons and its reduction in Parkinson’s disease (characterized by dopamine deficiency) [50], the increase in brain dopamine content following infusion of angiotensin 1–7 in the hypothalamus of rats [51], as well as dramatically low serotonin levels in the blood and the brain of ACE2-knockout mice [52]. SARS-CoV-2 is a neuroinvasive and neurotropic virus able to infect neural cells through binding of the ACE2 receptor [53]. Given that, upon infection, SARS-CoV-2 downregulates ACE2 [54], the defective expression of ACE2 might be paralleled by a DDC dysfunction, with consequent potentially altered neurotransmitter levels in the brain [43]. This mechanism could explain some of Post-COVID-19 condition’s neuropsychiatric manifestations such as anxiety, depression, and chronic fatigue [55]. Indeed, the alterations in dopamine and serotonin homeostasis are deeply involved in the development of fatigue [56,57].

On the other hand, there is extensive preclinical and clinical evidence that hypothalamic phospholipid liposomes increase monoaminergic neurotransmission, as shown by the activation of tyrosine hydroxylase (the rate-limiting enzyme in the synthetic pathway of dopamine and other catecholamines); the increase in monoamines turnover and release; the stimulation of the dopamine-dependent adenylyl cyclase and the increase in dopamine metabolite levels in human cerebrospinal fluid (CSF); the decrease in prolactin secretion through dopamine agonist activity (dopamine being the main inhibitor of the prolactin synthesis and release); and, finally, the modification of the receptor adaptation of central aminergic neurons to chronic treatment with antidepressants [47,48,58,59,60,61]. The monoaminergic effect of hypothalamic phospholipid liposomes renders them highly relevant for the treatment of fatigue, where a dopaminergic effect is particularly needed, as well as for the treatment of other COVID-19 neuropsychiatric sequalae, such as anxiety and depression.

#### 4.1.2. Neuroinflammation, Demyelination, and Impaired Neurogenesis

Other pathophysiological mechanisms potentially of great importance in PCC, such as neuroinflammation, demyelination, and impaired neurogenesis, have also been corroborated from animal model and human studies [23,27,62,63]. Even in mild COVID-19 infection, the inflammatory response caused by the respiratory COVID-19 induces neuroinflammation through CSF cytokine elevation and microglial reactivity [23,64]. Interleukins with antineurogenic effects such as IL-1β and IL-6 are particularly elevated in the brain of COVID-19 subjects, which leads to neuronal damage and impaired neurogenesis in structures such as the hippocampus, explaining learning, memory, and executive dysfunctions [64]. Neuroinflammation, probably in combination with other factors such as the direct effect of the virus on oligodendrocytes and cerebrovascular disorders, causes persistent loss of oligodendrocytes and demyelination [27,64]. Numerous reviews and theoretical and experimental studies convincingly indicate that demyelination may underlie many neuropsychiatric sequalae of COVID-19 [27].

Hypothalamic phospholipid liposomes exert a neurotrophic effect by improving the membrane structure and function, increasing endogenous phospholipid synthesis, and promoting dendritogenesis, as demonstrated by the increased density of dendritic spine [47,48]. Additionally, studies using animal models have demonstrated the antagonizing effect of phospholipids on demyelination, emphasizing the importance of phospholipid metabolism in myelination and myelin maintenance [65,66,67]. These studies suggest that phosphatidylcholine and phosphatidylethanolamine ameliorate myelination deficit [66], whereas phosphatidylserine prevents autoimmune demyelination [67]. Furthermore, recent preclinical studies have shown that hypothalamic phospholipid liposomes have a positive effect on hippocampal neurogenesis and an antagonizing effect on neuroinflammation [48]. Chronic treatment with hypothalamic phospholipid liposomes has been shown to reverse and prevent the reduction in neurogenesis induced by chronic stress in the dentate gyrus of the hippocampus in a study on rats [48]. Meanwhile, in a model of neuro-inflammation induced by lipopolysaccharide (LPS) injection, hypothalamic phospholipid liposomes antagonized, in a dose-dependent manner, the proinflammatory cytokine release elicited by LPS (IL-1β, IL-6, TNF-α) [47]. These effects on neuroplasticity and on the cytokines involved in COVID-induced neuroinflammation further strengthen hypothalamic phospholipid liposomes’ relevance in the treatment of PCC.

#### 4.1.3. Cerebral Hypometabolism

Case–control, cohort, and case studies using [18F] fluorodeoxyglucose positron emission tomography/computed tomography (18F-FDG-PET/CT) suggest that cerebral metabolic alterations may be responsible for the neurocognitive findings in PCC [68,69,70,71,72,73]. Hypometabolism of different brain areas (especially of the frontal cortex) in PCC patients suffering from neurocognitive symptoms is the main finding of these studies [72]. Hypothalamic phospholipid liposomes activate cerebral metabolism by increasing brain glucose content and phospholipid synthesis [47]. In addition, they are specifically indicated as adjuvant treatment in cerebral metabolic alterations caused by neuroendocrine disorders [48].

#### 4.1.4. Male Fertility Alterations

Low serum testosterone levels have been encountered in as much as 30% of men, up to 12 months after COVID-19 infection [74]. Low testosterone, in combination with other alterations such as psychological stress, activation of the HPA axis, and low dopamine levels, may contribute to erectile dysfunction and loss of libido [43,75,76,77]. Meanwhile, in male athletes, phosphatidylserine has been shown to increase plasma levels of testosterone compared to placebo [78] as well as increasing the testosterone to cortisol ratio in an exercise-related context [79]. Furthermore, it has been hypothesized that phospholipids increase the capacity of high-density lipoproteins (HDLs) to take up free cholesterol from the cytoplasm membrane of peripheral cells and to transport it in the esterified form to the steroid producing glands where it serves as a precursor to steroid hormones such as testosterone [80]. These effects, together with the normalizing effect on HPA axis and the dopaminergic effect of hypothalamic phospholipid liposomes [47], may account for their potential beneficial role in male sexual health alterations.

### 4.2. Clinical Evidence on Hypothalamic Phospholipid Liposomes and Its Implications for Post-COVID-19 Condition

Although there are currently no published clinical studies on the efficacy of hypothalamic phospholipid liposomes in PCC, the available evidence in other conditions supports their potential clinical relevance as a therapeutic option for PCC, in particular for symptoms such as anxiety and depression, chronic fatigue, brain fog, and potentially for orthostatic intolerance and male sexual health problems as well (Table 2).

Clinical evidence on the efficacy and safety of hypothalamic phospholipid liposomes (Liposom Forte^®^) has been generated in open studies with and without a control group, in drug-controlled trials and in double-blind, randomized, placebo-controlled trials [47]. Hypothalamic phospholipid liposomes, as an add-on treatment to antidepressant therapy, further improve depressive symptomatology while reducing antidepressant effect latency compared to antidepressant therapy alone [47,81,82,83]. In a double-blind, randomized, placebo-controlled trial, hypothalamic phospholipid liposomes in monotherapy were active against mild anxiety and depressive symptoms in menopausal women [84].

Furthermore, hypothalamic phospholipid liposomes have shown efficacy against other clinical symptoms that are commonly encountered in PCC. They improve asthenia caused by menopause [84] or induced by trazodone [83]. Additionally, hypothalamic phospholipid liposomes are effective against restlessness and dizziness [84]. Furthermore, hypothalamic phospholipid liposomes antagonized the hypotension and the reflex tachycardia caused by trazodone [83].

Taking into consideration that hypothalamic phospholipid liposomes contain different phospholipids, efforts have been made to identify the effect of the specific phospholipids in the mixture. Evidence during the initial phases of research suggested that phosphatidylserine might be the active ingredient of the mixture [85]. Phosphatidylserine is an essential component of the cerebral cortex and is associated with cognitive function [86]. In 2003, based on preliminary evidence, the FDA authorized a Qualified Health Claim that phosphatidylserine may reduce the risk of dementia and cognitive dysfunction in the elderly [87]. In a more recent meta-analysis, phosphatidylserine was shown to improve age-associated cognitive decline, especially in memory, with no adverse effects [86]. Furthermore, phosphatidylserine has been shown to benefit the memory of a small group of patients with Alzheimer’s disease [88]. These data suggest that phosphatidylserine may also display its clinical benefits against cognitive dysfunction caused by PCC, widely known as brain fog.

Additionally, preliminary evidence from an open clinical study has demonstrated that phospholipids (mainly phosphatidylcholine) may be useful and well tolerated in the treatment of male sexual disorders such as erectile dysfunction and loss of libido [80].

Considering the clinical relevance of ME/CFS and brain fog, as well as the substantial lack of specific pharmacotherapy, before considering drugs of the class of selective serotonin reuptake inhibitors (SSRIs) that could generate benefit in selected patients with ME/CFS [89], it seems reliable to try one or more treatment cycles with hypothalamic phospholipid liposomes, following a therapeutic regimen already proved to be safe and effective in previous clinical trials (one vial of Liposom Forte^®^ twice a day) [83,90]. Indeed, our initial clinical experience seems to corroborate the scientific rationale on hypothalamic phospholipid liposomes’ effectiveness in PCC. Additionally, hypothalamic phospholipid liposomes, by providing a rapid clinical improvement [47,48,81,82,83,84], may also decrease the excess costs of patients suffering from PCC, another important burden of this condition [91]. Nevertheless, it must be emphasized that pharmacotherapy should be part of an integrated multidisciplinary approach including treatments such as physical and neuro-cognitive rehabilitation [8].

Therefore, it is imperative to make any effort to collect more extensive and robust clinical data, observing a cohort of treated patients, evaluating them with a point-by-point questionnaire administered before and after therapy, and using an adequate follow up of at least 3–6 months. The urgency to find adequate responses for patients with PCC must not exempt itself from adopting the research methods required by evidence-based medicine.

**Table 2:** Post-COVID-19 condition’s pathophysiology and clinical manifestations matched to the relevant hypothalamic phospholipid liposomes’ mechanism of action and clinical evidence (ACE2—Angiotensin I Converting Enzyme 2; IL-1β—Interleukin 1β; IL-6—Interleukin 6; TNF-α—Tumor Necrosis Factor α; PE—Phosphatidylethanolamine; PC—Phosphatidylcholine; PS—Phosphatidylserine).

Post-COVID-19 Condition	Hypothalamic Phospholipid Liposomes
Pathophysiology	Mechanism of Action
Hypometabolic activity in certain brain areas [72]	Activation of cerebral metabolism (i.e., increased brain glucose content and phospholipid synthesis) [47]
ACE2–Dopa Decarboxylase co-expression, which leads to impaired monoaminergic neurotransmission [49]	Increased catecholamine turnover and release, stimulation of tyrosine hydroxylase and dopamine-dependent adenylyl cyclase, modification of monoaminergic receptor adaptation [47,48]
Neuroinflammation from CSF cytokine elevation (e.g., IL-1β, IL-6) and microglial reactivity [23,62,64]	Antagonizing effect on proinflammatory cytokines (IL-1β, IL-6, TNF-α) in different brain areas [47]
Demyelination and impaired neurogenesis [27,64]	Neurotrophic effect, increase in neurogenesis and dendritogenesis, as well as antagonizing effect of PE, PC, and PS on demyelination [48,66,67]
Low testosterone [74,77]	PS increases plasma levels of testosterone compared to placebo and the testosterone to cortisol ratio in an exercise-related context [78,79]
**Clinical Manifestations**	**Clinical Evidence**
Fatigue	Improvement of asthenia [83,84]
Brain fog	PS: Improves age-associated cognitive decline, especially memory, with no adverse effects [86]. May reduce the risk of dementia and cognitive dysfunction in the elderly [87]. Improved the memory of a small group of patients with Alzheimer’s disease [88].
Anxiety and depression	Improvement in the symptomatology of anxiety and depression as monotherapy or add-on to antidepressants [47,48]
Orthostatic intolerance	Antagonizing effect on hypotension and reflex tachycardia caused by trazodone [83]
Male sexual health problem	Phospholipids (PC in particular) improve erectile dysfunction and loss of libido [80]

## 5. Conclusions

Post-COVID-19 condition is a heterogeneous clinical condition in which ME/CFS and brain fog stand out among the different clinical symptoms and syndromes. Cerebral metabolic alterations and neuroendocrine disorders seem to constitute an important part of Post-COVID-19 condition. Given the substantial lack of specific drugs and effective therapeutic strategies, hypothalamic phospholipid liposomes, which have been on the market for several years as adjuvant therapy of cerebral metabolic alterations resulting from neuroendocrine disorders, can be taken into consideration in an overall therapeutic strategy that aims to control Post-COVID-19 condition–associated symptoms and syndromes. The pharmacological mechanisms and clinical effects of hypothalamic phospholipid liposomes strongly support their potential effectiveness in Post-COVID-19 condition. Our initial clinical experience corroborates this rationale. Further clinical research is needed in order to obtain sufficient evidence on the role of hypothalamic phospholipid liposomes as an adjuvant treatment for some manifestations of Post-COVID-19 condition.

## Data Availability

No new data were created or analyzed in this study. Data sharing is not applicable to this article.
